# Comparative Evaluation of Cameroonian Honey and Normal Saline in the Management of Chronic Wounds: A Randomized Controlled Trial

**DOI:** 10.1002/hsr2.71719

**Published:** 2026-01-06

**Authors:** Bih Vanessa Tita, Chi Solange Ika, Ngwa Fabrice Ambe, Binwi Florence Endeley, Palle John Ngunde

**Affiliations:** ^1^ Department of Nursing, Faculty of Health Sciences University of Buea Buea Cameroon; ^2^ Department of Medical Laboratory Science, Faculty of Health Sciences University of Buea Buea Cameroon

**Keywords:** Cameroonian honey, chronic wounds, normal saline, wound healing, wound management

## Abstract

**Background and Aim:**

In Cameroon, honey shows potential as a low‐cost, effective wound treatment due to its antimicrobial and wound‐healing properties. This study seeks to evaluate the clinical efficacy of Cameroon honey compared to normal saline in the management of chronic wounds at the Buea Regional Hospital, Cameroon.

**Methods:**

This hospital‐based randomized controlled trial was conducted at Buea Regional Hospital, Cameroon, over 6 months. Eighteen patients with chronic wounds were randomly assigned to receive either honey (*n* = 9) or normal saline (*n* = 9) dressings. Honey‐treated wounds were dressed with unprocessed Cameroonian honey, while saline‐treated wounds received saline applications. Wound size, granulation tissue formation, infection rates, and pain were monitored over 12 weeks. Wound swabs were collected and inoculated on blood and MacConkey Agar. Isolates were identified using API 20. Data were analyzed using SPSS, and statistical significance was *p* < 0.05.

**Results:**

Honey‐treated wounds achieved a significantly higher wound closure rate (97%) and granulation tissue formation (90%) compared to saline‐treated wounds (63% and 70%, respectively; *p* < 0.001). By Week 12, no bacteria were isolated from the honey group, while saline‐treated wounds harbored *Staphylococcus aureus, Pseudomonas aeruginosa*, and *Staphylococcus saprophyticus*. Pain, exudate, and inflammation were reduced faster in the honey group, with higher patient satisfaction.

**Conclusion:**

Honey is a superior alternative to normal saline for managing chronic wounds, offering improved healing outcomes, effective infection control, and enhanced patient satisfaction. Its integration into routine wound care in resource‐limited settings like Cameroon is highly recommended.

**Relevance to Clinical Practice:**

This study explores the potential of Cameroonian honey to promote quicker wound healing and improve the quality of life for patients with chronic wounds. By offering a low‐cost, accessible alternative to normal saline, it supports better outcomes in resource‐limited settings.

**What does this paper contribute to the wider global clinical community?**

- The findings contribute to global evidence on natural therapies, supporting the integration of locally sourced solutions like honey into wound care practices.
- They highlight the potential of such remedies to accelerate healing, reduce complications, and improve patient well‐being.

**Patient Contribution:**

Patients participated as the primary subjects in the study, consenting to receive either Cameroonian honey or normal saline for the dressing of their chronic wounds. Through their involvement, they provided firsthand data on wound healing progress and any side effects or improvements experienced.

## 1 Introduction

Chronic wounds are a major public health issue worldwide, disproportionately affecting individuals in low‐ and middle‐income countries (LMICs). These wounds, defined as wounds that fail to progress through normal stages of healing within 3 months, include venous ulcers, diabetic foot ulcers, pressure ulcers, and arterial ulcers. Chronic wounds not only cause physical and psychological distress to patients but also present a significant economic burden to healthcare systems globally, with costs reaching up to $96.8 billion annually in developed countries alone [1, 2]. However, in resource‐limited settings such as sub‐Saharan Africa, the prevalence and management of chronic wounds remain under‐researched, exacerbating challenges in delivering effective care [3]. In Cameroon, the burden is intensified by limited access to advanced wound care materials, insufficient healthcare resources, and the coexistence of communicable and non‐communicable diseases that complicate wound healing [4, 5].

The management of chronic wounds in LMICs often relies on basic wound care practices, with normal saline being the most widely used agent for cleaning and dressing wounds. Normal saline (0.9% sodium chloride solution) is favored for its isotonic nature, availability, and safety, as it does not cause cellular damage or irritation [6]. However, its lack of antimicrobial properties limits its effectiveness in addressing one of the major challenges of chronic wounds: infection [7]. Chronic wounds are frequently colonized by pathogenic bacteria such as *Staphylococcus aureus, Pseudomonas aeruginosa*, and *Escherichia coli*, which delay healing and increase the risk of complications, including systemic infections and sepsis [8, 9].

Honey, a natural substance with a long history of medicinal use, has emerged as a promising alternative for chronic wound care. Ancient civilizations, including those in Africa, used honey to treat wounds due to its antimicrobial, anti‐inflammatory, and wound‐healing properties [10, 11]. In recent years, honey has gained renewed attention in modern medicine as a scientifically validated option for wound management. Studies have shown that honey promotes wound healing by creating a moist environment, accelerating tissue regeneration, and exhibiting broad‐spectrum antimicrobial activity [12]. These effects are attributed to its unique chemical composition, which includes high sugar content, low pH, and the production of hydrogen peroxide through the enzymatic action of glucose oxidase [13].

The antimicrobial properties of honey are particularly significant in the context of rising antibiotic resistance. Research has demonstrated that honey is effective against antibiotic‐resistant bacteria, including methicillin‐resistant *Staphylococcus aureus* (MRSA) and vancomycin‐resistant *Enterococci* (VRE) [14, 15]. In Africa, where traditional medicine remains an integral part of healthcare, honey is widely available and culturally accepted, making it a cost‐effective alternative to synthetic wound care products [16]. For instance, studies in Ghana and Kenya have highlighted honey's efficacy in reducing bacterial load, accelerating wound closure, and improving patient outcomes [17, 18]. A study conducted in Nigeria demonstrated that honey‐treated wounds achieved significantly higher granulation tissue formation and wound closure rates compared to wounds treated with saline [19]. Similar findings were reported in South Africa, where honey reduced bacterial colonization and improved wound healing outcomes in diabetic foot ulcers [20]. These studies suggest that honey has the potential to address critical gaps in wound care in resource‐limited settings like Cameroon, where infections and delayed healing are common challenges.

In Cameroon, the traditional use of unprocessed, multifloral honey is well‐established. However, scientific studies remain limited. A chemical analysis of Ngaoundere honey via GC‑MS identified fourteen bioactive compounds, including 5‑hydroxymethyl‑2‑furancarboxaldehyde (~36%), 2‑butoxyethyl acetate (~11%), 2,4‑dimethyl‑1‑pentanol (~9%), and 3,5‑dihydroxy‑6‑methyl‑2,3‑dihydro‑4H‑pyran‑4‑one (~8.9%), which correlate with antimicrobial and antioxidant potential [21]. In a nationwide study of Cameroonian honeys, including samples from the Sudano‑Guinean and Guinea‑savannah zones encompassing Ngaoundere, physicochemical characterization revealed multifloral honeys with densities of 1.43–1.51 g/mL, sugar content of 70.3%–83.2%, and acidic pH values of 3.30–4.10. Total phenolic content ranged from 26.8 to 85.1 mg GAE/100 g, and flavonoid content from 5.2 to 14.5 mg QE/100 g. Darker honeys and those with pH around 4 demonstrated stronger antioxidant activity (DPPH, FRAP, TAC assays) [22].

Prior in vitro work also examined antibacterial activity of honey from Oku, Mbengwi, and Ngaoundere against clinical isolates of *S. aureus* and *E. coli*, showing MICs from 1.5% to 49% v/v and effective bacterial inhibition at ≥ 40% concentration, comparable to ciprofloxacin at ≥ 60% (pH ≈ 5) [23]. When compared to other honeys, Cameroonian honey demonstrates comparable or superior antimicrobial properties. For example, Malaysian Tualang honey, though effective, often requires a higher concentration to achieve similar inhibition zones [24]. Manuka honey, known for methylglyoxal (MGO), shows strong biofilm disruption [25], but Cameroonian honey's polyphenol‐rich profile provides broader antioxidant support [22]. Another study comparing Cameroonian honeys to medical‐grade Manuka found MICs of ~10% w/v for both honeys against *E. coli, P. aeruginosa*, and *S. aureus*, and demonstrated notable non‑peroxide antimicrobial activity tied to antioxidant and phenolic content [26].

Despite these rich in vitro data, no clinical trial has yet evaluated the efficacy of Cameroonian (Ngaoundere) honey in chronic wound management. Moreover, concerns around sterility and pollen presence in raw, unprocessed honey, critical for clinical use, remain unaddressed. While raw honey naturally contains pollen, which aids in floral identification but may pose allergenic risks, it also retains important bioactive compounds. In previous Cameroonian studies, low microbial counts were noted in honey despite a lack of sterilization, likely due to low moisture, high sugar content, and acidity. Nonetheless, for medical applications, filtration or gamma irradiation is recommended to ensure sterility while preserving bioactive efficacy [27].

This study seeks to evaluate the clinical efficacy of Cameroon honey compared to normal saline in the management of chronic wounds at the Buea Regional Hospital, Cameroon. By investigating key wound‐healing parameters such as wound size reduction, granulation tissue formation, pain relief, infection control, and patient satisfaction, this research aims to provide evidence for the integration of honey into routine wound care protocols. Given the socio‐economic and healthcare challenges in Cameroon, promoting the use of honey could lead to improved patient outcomes, reduced healthcare costs, and a reduction in antibiotic use and resistance. Furthermore, this study contributes to the growing body of evidence supporting the role of natural remedies in modern medicine and highlights the importance of context‐specific interventions for enhancing healthcare delivery in Africa.

## 2 Materials and Methods

### 2.1 Study Design and Area

This study was a hospital‐based, randomized controlled trial conducted at the Buea Regional Hospital, Cameroon, over 6 months. The aim was to compare the clinical efficacy of Cameroonian honey to normal saline in the management of chronic wounds.

### 2.2 Study Population

The study population included adult patients aged 18 years and above presenting with chronic wounds of at least 3 months' duration.

#### 2.2.1 Inclusion Criteria

- Patients with chronic wounds hospitalized during the time of the study.
- Individuals who provided informed written consent to participate.

#### 2.2.2 Exclusion Criteria

- Patients with wounds resulting from malignancy (e.g., cancerous ulcers).
- Patients with a known history of pollen grain allergy.

### 2.3 Sample Size and Sampling Technique

The study recruited 18 participants using a convenience sampling method, based on the availability of patients meeting the inclusion criteria. Participants were randomly assigned to one of two groups:

1. Honey dressing group (*n* = 9).
2. Normal saline dressing group (*n* = 9).

Randomization was performed using a computer‐generated randomization sequence to ensure unbiased allocation.

### 2.4 Honey Origin and Characterization

The honey was sourced from local beekeepers in Ngaoundere (Adamawa Region, Northern Cameroon). The exact harvest date was not recorded. However, samples were harvested during the dry season (November) when local savannah flora is in bloom. It was a single multifloral, typical of Sudano‐Guinean savannah flora, including Terminalia and Syzygium nectar sources. It was collected using traditional techniques and filtered through sterile gauze to remove debris and stored at ambient temperature. Although direct measurements were not done in our lab, literature values for physico‐chemical properties include moisture (15.9%–21.4%), pH (3.3–4.1), density (1.39–1.51 g/mL), sugar content (70%–83%), phenolic content (26.8–85.1 mg GAE/100 g), and flavonoids (5.2–14.5 mg QE/100 g)^+^ [22]. GC‐MS analysis of Ngoaundere honey revealed major volatile compounds associated with antimicrobial and antioxidant activities, including 5‑hydroxymethyl‑2‑furancarboxaldehyde (~36%), 2‑butoxyethyl acetate ( ~11%), 2,4‑dimethyl‑1‑pentanol (~9%), and 3,5‑dihydroxy‑6‑methyl‑2,3‑dihydro‑4H‑pyran‑4‑one ( ~ 8.9%) [21].

### 2.5 Intervention Protocol

1. Honey dressing group:
  Material: unprocessed Cameroonian honey (from Ngaoundere).
  Procedure: Wounds were cleaned with sterile water, and honey was applied directly to the wound bed. The wounds were then covered with sterile gauze. Dressings were changed daily or as needed based on the amount of exudate.
2. Normal saline dressing group:
  Material: sterile normal saline (0.9% NaCl).
  Procedure: wounds were cleaned with sterile water, and gauze moistened with normal saline was applied to the wound. The wounds were then covered with sterile gauze. Dressings were also changed daily or as needed.

### 2.6 Outcome Measure

The primary outcome measure in this study was wound size reduction, assessed weekly using a sterile caliper. The reduction in wound size was calculated as a percentage of the initial wound area, providing an objective measure of the healing progress. Secondary outcomes included granulation tissue formation, which was visually evaluated as the percentage of the wound bed covered by granulation tissue, and wound closure percentage, calculated as the change in wound area over time.

Other secondary outcomes included the amount and type of wound exudate, recorded qualitatively as heavy, moderate, light, mild, or minimal, and purulent or serous. The presence of odour is categorized as foul, mild, or none. Pain levels were assessed weekly using a Visual Analog Scale (VAS), ranging from 0 (no pain) to 10 (worst possible pain). Signs of infection, such as redness, swelling, warmth, and pus, were recorded throughout the study.

To monitor systemic inflammation and bacterial colonization, C‐reactive protein (CRP) levels were measured from blood samples at Weeks 1, 6, and 12, and bacterial growth was evaluated through wound swabs taken at the same intervals. Finally, patient satisfaction was recorded at Week 12 using a qualitative scale, ranging from “very unsatisfied” to “very satisfied,” to capture participants' perceptions of their treatment outcomes.

### 2.7 Laboratory Analysis

#### 2.7.1 Wound Swab Collection

To collect a wound swab, the wound was cleaned with sterile normal saline to remove contaminants, and then a sterile swab was used to collect exudate from a 1 cm² area of viable tissue, avoiding necrotic areas. Levine technique was employed by rotating the swab while applying gentle pressure for about 5 s. The swabs were placed in a sterile container immediately after collection and labelled with the patient's information, and transported to the laboratory promptly to ensure accurate microbiological analysis. Wound swabs were obtained in three phases (Weeks 1, 6, and 12) to monitor bacterial colonization and infection.

#### 2.7.2 Culture

MacConkey Agar, mannitol salt agar, and blood agar were prepared according to the manufacturer's instructions. Each swab was plated onto blood agar, McConkey Agar and mannitol salt agar and incubated aerobically at 37°C for 24 h. All positive cultures were then identified at the species level by their colony characteristics, Gram‐staining reaction, and biochemical testing using the standard microbiological technique (colony characteristics, Gram staining, and biochemical testing). Gram‐positive cocci were identified using catalase and coagulase tests, while for the Gram‐negative bacilli, oxidase test, catalase test, and API20E were used for species identification.

### 2.8 Data Collection

Baseline socio‐demographic characteristics were collected using a structured questionnaire. Wound assessment data were collected weekly using a modified Bates‐Jensen Wound Assessment Tool [28]. Wound swabs were obtained in three phases (Weeks 1, 6, and 12) to monitor bacterial colonization and infection.

### 2.9 Data Analysis

Data were entered into Microsoft Excel and analyzed using SPSS version 16. Descriptive statistics summarized demographic and clinical data. Independent *t*‐tests (2‐sided) were used to compare mean values between the two groups for continuous variables. A *p* value of less than 0.05 was considered statistically significant.

### 2.10 Ethical Consideration

Ethical approval was obtained from the Institutional Review Board of the Faculty of Health Sciences, University of Buea (Approval No. 1826‐05). Additional authorization was secured from the Regional Delegation of Public Health, Southwest Region, to conduct the study (No. 422/427), and the Director of Buea Regional Hospital. Written informed consent was obtained from all participants before enrollment. Participants were assured of confidentiality, voluntary participation, and the right to withdraw from the study at any time without repercussions.

## 3 Results

### 3.1 Socio‐Demographic Characteristics

A total of 18 participants were included in the study, with 9 patients each in the honey and saline groups. Respondents were 18 years and above, with mean age of 39 years. The majority of the participants were 18–38 years old (56%), male (67%), married (61%), had a post‐secondary level of education (44%) (Table 1).

**Table 1:** Socio‐demographic characteristics of participants.

Variables	Categories	Frequency (*N*)	Percentage %
Age (in years)	18–38	10	56
	39–59	6	33
	60 and above	2	11
	Total	18	100
Gender	Female	6	33
	Male	12	67
Marital status	Total	18	100
	Married	11	61
	Widow (er)	3	17
	Total	18	100
Level of education	Primary	3	17
	Secondary	6	33
	Post‐secondary	8	44
	No Formal Education	1	06
	Total	18	100
Religion	Islam	4	22
	Christianity	14	78
	Total	18	100

### 3.2 Evaluation of the Effect of Honey and Saline Dressing on Wound Size

**Figure 1:**
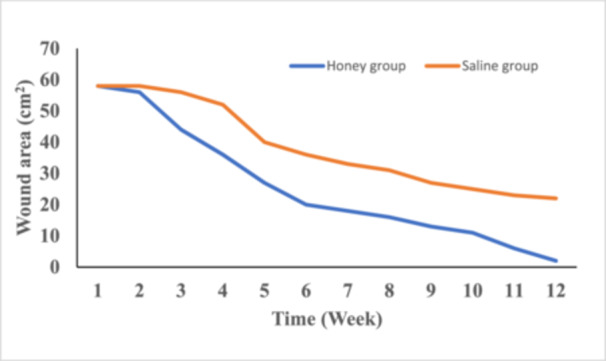
Change in wound size weekly.

The reduction in wound size was significantly greater in the honey group compared to the saline group. At Week 1, the average wound size was similar in both groups, with each group having a wound area of 58 cm². By week 12, the average wound size had reduced to 2 cm² in the honey group, representing a 97% closure rate, while the saline group achieved only a 63% closure rate, with an average wound size of 22 cm² (*p *< 0.001). These findings are illustrated in Figure 1, which shows the trend of wound size reduction over time.

### 3.3 Evaluation of the Effect of Honey and Saline Dressing on Bacterial Growth and C‐Reactive Protein Levels

The honey group showed complete elimination of bacterial growth by Week 12, with no organisms isolated. In contrast, the saline group continued to show bacterial colonization, including *S. aureus, P. aeruginosa*, and *Staphylococcus saprophyticus*, even at Week 12 (Table 2).

**Table 2:** Wound healing outcomes assessed by a modification of the Bates–Jensen wound assessment tool at Week 1, 6, and 12 (independent *t*‐test).

Variables	Groups	Week 1	Week 6	Week 12	*p* value
Wound closure %	Honey	3 ± 0.5	53 ± 5	97 ± 0.5	< 0.001
	Saline	0	31 ± 5	63 ± 3	
Exudate amount and type	Honey	Heavy, purulent	Light, serous	Minimal, serous	
	Saline	Heavy, purulent	Moderate, serous	Mild, serous	
% Granulation	Honey	20 ± 5	50 ± 10	90 ± 5	0.001
	Saline	20 ± 5	35 ± 10	70 ± 10	
Odour	Honey	Foul	None	None	
	Saline	Foul	Mild	None	
Pain (0–10)	Honey	7 ± 1	3 ± 1	0.5 ± 1	
	Saline	7 ± 1	5 ± 1	1 ± 1	
Signs of infection	Honey	Redness, swelling, warmth, pus	Mild redness	None	
	Saline	Redness, swelling, warmth, pus	Mild redness and swelling	None	
Bacteria isolated	Honey	*Escherichi coli*, *Staphylococcus epidermidis*, *Staphylococcus aureus*, *Pseudomonas aeruginosa*, *Staphylococcus saprophyticus*.	*Staphylococcus aureus, Pseudomonas aeruginosa*,	Nil	
	Saline	*Escherichi coli*, *Staphylococcus epidermidis*, *Staphylococcus aureus*, *Pseudomonas aeruginosa*, *Staphylococcus saprophyticus*.	*Staphylococcus aureus, Escherichi coli, Pseudomonas aeruginosa, Staphylococcus saprophyticus*	*Staphylococcus aureus, Pseudomonas aeruginosa, Staphylococcus saprophyticus*	

*Note:* Each value is expressed as mean ± standard deviation.

**Figure 2:**
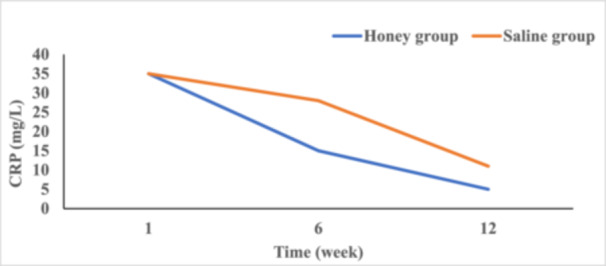
Change in CRP at Week 1, 6, and 12.

C‐reactive protein (CRP) levels, an indicator of systemic inflammation, were also significantly lower in the honey group compared to the saline group. At Week 1, the average CRP levels were 35 mg/L in both honey group and saline group. By Week 12, CRP levels had reduced to 5 mg/L in the honey group, compared to 11 mg/L in the saline group, indicating faster resolution of inflammation in the honey‐treated wounds (Figure 2).

### 3.4 Evaluation of the Effect of Honey and Saline Dressing on Wound Healing Outcomes and Patient Satisfaction

Wound healing outcomes, including granulation tissue formation, pain reduction, and exudate resolution, were superior in the honey group. Granulation tissue formation in the honey group reached 90% by Week 12, compared to 70% in the saline group (*p *= 0.001). Pain levels decreased more rapidly in the honey group, with participants reporting an average pain score of 0.5/10 by Week 12, compared to 1/10 in the saline group. Exudate levels, initially heavy in both groups at Week 1, reduced to minimal levels by Week 12 in the honey group, while mild levels persisted in the saline group (Table 2).

**Figure 3:**
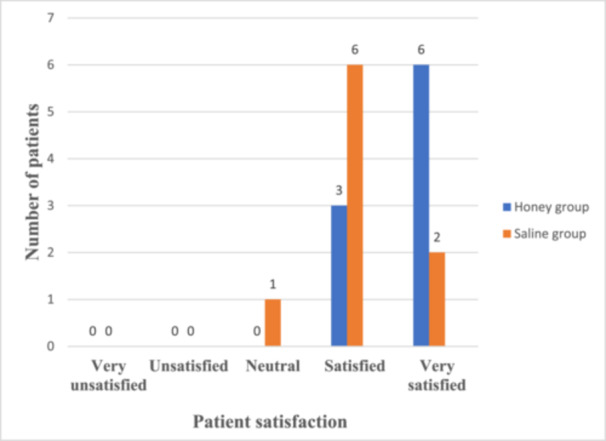
Assessment of patient satisfaction at Week 12.

Patient satisfaction was notably higher in the honey group. By Week 12, most participants in the honey group rated their experience as “very satisfied,” compared to “satisfied” ratings in the saline group (Figure 3).

## 4 Discussion

This study highlights the remarkable clinical effectiveness of honey, particularly unprocessed Cameroonian honey, in managing chronic wounds. When compared to normal saline, honey showed significant benefits, including faster wound size reduction, enhanced granulation tissue formation, better pain relief, improved infection control, and higher patient satisfaction. These findings highlight honey as not only a cost‐effective and accessible option but also as a scientifically validated alternative for chronic wound care, particularly in resource‐limited settings like Cameroon. Its unique properties make it an excellent candidate for routine wound care, offering a potential shift in treatment paradigms, particularly in settings where traditional medical resources are scarce [29, 30].

Wound size reduction and granulation tissue formation are crucial indicators of healing. In this study, wounds treated with saline achieved a 63% closure rate with tissue granulation reaching 70% by Week 12, with the average wound size reducing from 58 cm^2^ at Week 1–22 cm^2^ at Week 12. These outcomes demonstrate the ability of saline to support basic wound healing by maintaining a moist wound environment that promotes epithelialization and fibroblast migration [1, 10]. In contrast, honey‐treated wounds achieved a 97% closure rate and 90% granulation by Week 12. The faster wound size reduction in the honey group from 58 cm^2^ at Week 1–2 cm^2^ at Week 12, highlights honey's superior ability to accelerate tissue regeneration. These outcomes are consistent with findings from various studies showing honey's effectiveness in wound healing [29, 30]. Granulation tissue formation is driven by the activity of fibroblasts, angiogenesis, and extracellular matrix deposition, particularly collagen [31]. Honey‐treated wounds could achieve a faster wound size reduction and tissue granulation because, in addition to providing a moist healing environment that enhances fibroblast proliferation and keratinocyte migration [32], honey possesses unique properties that support the healing process. Honey's composition, particularly the presence of hydrogen peroxide, plays a critical role in promoting angiogenesis. Unlike higher concentrations of hydrogen peroxide, which can be cytotoxic, the low, controlled release of hydrogen peroxide from honey supports the formation of new blood vessels without damaging tissue [29]. This is particularly important for chronic wound healing, as angiogenesis ensures the delivery of essential nutrients and oxygen to the wound bed, accelerating recovery [30].

A major attribute of honey, especially unprocessed, Cameroonian honey, is its strong antimicrobial activity, which is absent in saline [7]. By Week 12, no bacteria were isolated from the honey‐treated wounds, while the saline‐treated group continued to harbor pathogens like *S. aureus, P. aeruginosa*, and *S. saprophyticus*. The antimicrobial efficacy of honey is multifaceted and attributed to several mechanisms: the high sugar content in honey creates a hyperosmolar environment that dehydrates and lyses bacterial cells; the enzyme glucose oxidase in honey catalyzes the production of hydrogen peroxide, which acts as a mild antiseptic; honey's naturally low pH (3.2–4.5) inhibits the growth of many pathogens by disrupting microbial enzymatic activity [9, 12, 22, 29, 31, 32, 33]. Also, unprocessed honey, such as that used in this study, retains a higher concentration of bioactive compounds that are often lost in processed honey. These include natural antimicrobial peptides, phenolics, and antioxidants, which can enhance its effectiveness in combating both common and antibiotic‐resistant bacteria like Methicillin‐resistant *Staphylococcus aureus* (MRSA) and Vancomycin‐resistant Enterococci (VRE) [14, 15, 21, 22, 29]. Moreover, the natural floral biodiversity of the Adamawa highlands, where this honey originates, likely contributes to its unique bioactive composition, setting it apart from mono‐floral honeys like Manuka [21]. The absence of antimicrobial properties in saline makes dressings with saline overly reliant on adjunctive antibiotic medications, which contribute to the issue of antibiotic resistance and prolong treatment [7, 8]. In settings like Cameroon, where the burden of antibiotic resistance and high cost of wound care is a growing concern, honey provides an invaluable alternative to traditional antibiotics, offering a sustainable and effective solution for wound infections.

Another major advantage of honey lies in its anti‐inflammatory properties, which contribute to faster pain relief and reduced systemic inflammation. Chronic wounds are often characterized by prolonged inflammation, a barrier to healing. Honey‐treated wounds demonstrated significant reductions in C‐reactive protein (CRP) levels, from 35 mg/L at Week 1–5 mg/L by Week 12, compared to 11 mg/L in the saline group. Honey achieves this by modulating inflammatory response by reducing pro‐inflammatory cytokines such as tumor necrosis factor‐alpha (TNF‐α), interleukin‐6 (IL‐6), and interleukin‐1 beta (IL‐1β), while promoting the release of anti‐inflammatory cytokines like interleukin‐10 (IL‐10) [11, 30]. This modulation of the immune response not only promotes healing but also alleviates the pain and discomfort commonly associated with chronic wounds [6]. Additionally, the moist healing environment created by honey prevents desiccation of the wound bed, further contributing to pain reduction [11, 31]. This could be reflected in lower pain scores in the honey group (0.5/10) compared to the saline group (1.0/10) by week 12.

Honey provided a more satisfactory treatment experience than saline. Patients treated with honey reported being “very satisfied” with their care, citing faster healing, reduced pain, and absence of odour as key factors. The slow, controlled release of hydrogen peroxide from honey also minimizes irritation compared to conventional antiseptics, which can cause tissue damage and discomfort. Both honey and saline reduced exudate and odour, but honey demonstrated faster and more complete resolution. By Week 12, honey‐treated wounds exhibited minimal exudate and no odour, whereas mild levels persisted in the saline group. Excessive exudate and malodour are often indicative of bacterial colonization and tissue degradation [34]. Honey's ability to manage wound exudate and odour was another significant finding in this study, underscoring honey's role in controlling bacterial growth and tissue necrosis [29]. The antimicrobial properties of honey eliminate the causative pathogens, while its anti‐inflammatory effects reduce vascular permeability and exudate production, contributing to better wound management [32]. These factors together enhance patient comfort and improve the overall healing experience. In contrast, patients in the saline group reported slower progress and moderate satisfaction, reflecting the limited impact of saline on infection and inflammation.

Honey's effectiveness in wound healing, combined with its ease of use and minimal side effects, makes it a highly acceptable treatment option, especially in African settings where it is culturally accepted and traditionally used in medicinal practices [31]. In addition, the natural composition of Cameroonian honey ensures that it aligns with local preferences, making it a sustainable and culturally appropriate solution for wound care [33].

This study had a few limitations. The small sample size and single‐center design may limit the generalizability of the findings. Further research with larger sample sizes and multiple centers is needed to validate the results and expand the evidence base for using honey in chronic wound care. Also, this study did not include NMR certification or palynological analysis to confirm the purity and botanical origin of the honey used, but relied on reports from local beekeepers, which limits conclusions about its exact composition. Further studies should incorporate these advanced analyses to better characterize the honey and validate its therapeutic potential.

## 5 Conclusion

While both Cameroonian honey and saline play important roles in chronic wound management, honey's multifunctional properties make it a superior option. Its ability to address infection, inflammation, and tissue regeneration simultaneously highlights its transformative potential for wound care, particularly in resource‐limited settings like Cameroon. These findings strongly support the integration of honey into routine wound care protocols, offering a sustainable, cost‐effective, and culturally appropriate solution for managing chronic wounds. However, further research is essential to optimize its use, ensure standardization, and explore its application in broader clinical contexts.

### 5.1 Relevance to Clinical Practice

This study is relevant to clinical practice as it explores the potential of Cameroonian honey to promote quicker wound healing and improve the quality of life for patients suffering from chronic wounds; a condition that often leads to prolonged pain, immobility, and increased risk of infection. By comparing honey to normal saline in a randomized controlled trial, the study provides evidence that can guide clinicians in selecting effective, affordable treatment options. Cameroonian honey, being locally available and cost‐effective, presents a promising alternative for enhancing wound healing outcomes in resource‐limited settings where access to advanced wound care products may be restricted. This can lead to shorter healing times, reduced healthcare costs, and improved patient satisfaction, ultimately supporting more equitable and sustainable healthcare delivery.

## Author Contributions

**Bih Vanessa Tita:** conceptualization, methodology, formal analysis, writing – original draft, writing – review and editing, software. **Chi Solange Ika:** conceptualization, data curation, methodology, writing – review and editing. **Ngwa Fabrice Ambe:** methodology, data curation, validation, formal analysis, writing – review and editing, software. **Binwi Florence Endeley:** conceptualization, methodology, supervision, writing – review and editing, project administration. **Palle John Ngunde:** methodology, supervision, project administration, writing – review and editing, validation.

## Funding

The authors received no specific funding for this work.

## Ethics Statement

Ethical approval was obtained from the Institutional Review Board of the Faculty of Health Sciences, University of Buea (Approval No. 1826‐05). Additional authorization was secured from the Regional Delegation of Public Health, Southwest Region (No. 422/427), and the Director of Buea Regional Hospital. Written informed consent was obtained from all participants before enrollment. Participants were assured of confidentiality, voluntary participation, and the right to withdraw from the study at any time without repercussions.

## Conflicts of Interest

The authors declare no conflicts of interest.

## Transparency Statement

The lead author, Bih Vanessa Tita, affirms that this manuscript is an honest, accurate, and transparent account of the study being reported; that no important aspects of the study have been omitted; and that any discrepancies from the study as planned (and, if relevant, registered) have been explained.

## Data Availability

The data that support the findings of this study are available from the corresponding author upon reasonable request.
